# Near-Surface Hydrogen Species Tune the Selectivity of Chemical Reactions on Metal Oxide Surfaces

**DOI:** 10.1021/prechem.5c00060

**Published:** 2025-08-01

**Authors:** Yi-Chun Chu, Weixin Huang, Xin-Ping Wu, Xue-Qing Gong

**Affiliations:** † State Key Laboratory of Green Chemical Engineering and Industrial Catalysis, Centre for Computational Chemistry and Research Institute of Industrial Catalysis, School of Chemistry and Molecular Engineering, 627273 East China University of Science and Technology, Shanghai 200237, P.R. China; ‡ State Key Laboratory of Precision and Intelligent Chemistry, iChEM, Key Laboratory of Surface and Interface Chemistry and Energy Catalysis of Anhui Higher Education Institutes, Department of Chemical Physics, 12652 University of Science and Technology of China, Hefei 230026, P.R. China; § State Key Laboratory of Synergistic Chem-Bio Synthesis, School of Chemistry and Chemical Engineering, 12474 Shanghai Jiao Tong University, Shanghai 200240, P.R. China

**Keywords:** Ga_2_O_3_, Catalysis, Hydride, Oxygen Vacancy, Density Functional Theory Calculations

## Abstract

Upon interaction with H_2_, metal oxides can be reduced. Such a reduction may consequently alter their interactions as well as the evolution of hydrogen species on metal oxide surfaces. However, this has not been thoroughly and precisely studied on the atomic scale. Accordingly, systematic density functional theory (DFT) calculations were performed on a representative metal-oxide catalyst surface, namely β-Ga_2_O_3_(100). It was found that oxygen vacancy clusters can readily form upon reduction and that such vacancy clusters enable the infiltration of surface hydrogen species into the near-surface region. The calculated relative population of near-surface hydrogen species reaches approximately 5% under typical experimental conditions for hydrogenation and dehydrogenation reactions on Ga_2_O_3_ surfaces, and thus, these species cannot be ignored. To study the effect of near-surface hydrogen species on the surface chemistry of metal oxides, two important reactions were considered. The first is H_2_ dissociation, which is an important process in catalytic hydrogenation (and dehydrogenation) reactions. The second is CO_2_ hydrogenation, which is a representative hydrogenation reaction. It was found that the presence of near-surface hydrogen species results in modulation of the surface electronic and chemical properties of β-Ga_2_O_3_(100), leading to a change in the preferred pathway for these surface chemical reactions. These findings emphasize the crucial role of near-surface hydrogen species in tuning the selectivity of chemical reactions on metal oxide surfaces. This perspective has not been identified thus far, to the best of our knowledge, but is consistent with previously reported experimental results in many aspects.

## Graphical abstract

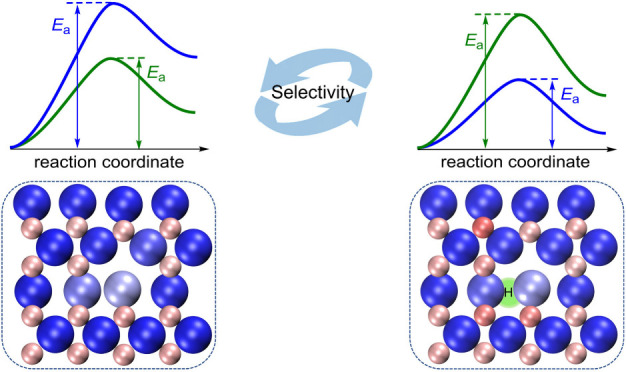

## 1 Introduction

Hydrogen species, including proton (H^+^) and hydride (H^–^) and hydrogen radical (H·), are commonly present on metal oxide surfaces.^1–3^ These species may be involved in catalytic processes.^4,5^ In particular, hydride species are highly reactive in hydrogenation and dehydrogenation reactions catalyzed by metal oxide surfaces, stabilizing specific transition states and intermediates.^1,5–8^ However, hydride species are often less stable than other hydrogen species, such as a proton.^5,9,10^ To stabilize hydrides on metal oxide surfaces, a sufficient concentration of oxygen vacancies and/or low-coordinated metal cations are often required.^11,12^

Among various metal oxides, β-Ga_2_O_3_ is a material of growing interest as a catalyst.^13–17^ Previous theoretical studies^18,19^ have shown that hydrides are rather stable on the (100) surface of β-Ga_2_O_3_, making it an ideal metal oxide surface for producing hydride species to facilitate specific catalytic reactions. Chen et al.^20^ employed solid-state nuclear magnetic resonance (ssNMR) to demonstrate the presence of hydride species during propane dehydrogenation (PDH) on the Ga_2_O_3_ catalyst. A few recent studies have emphasized the crucial role of hydride species on the surfaces of β-Ga_2_O_3_ in converting alkanes into olefins.^21,22^ These studies highlight the significant role of surface hydride species in β-Ga_2_O_3_-catalyzed reactions.

To effectively generate surface hydrogen species, metal oxide catalysts are often required to be exposed to a hydrogen atmosphere. The interaction between H_2_ and metal oxide surfaces can create oxygen vacancies on the surfaces and/or in the near-surface regions.^2,23–25^ It can be expected that such a reduction process can in turn alter the interaction between H_2_ and metal oxide surfaces as well as the evolution of surface hydrogen species. However, such issues have been less investigated to date. In particular, it is important to understand how the surface structure and properties change upon interaction with hydrogen, and what effect this has on the chemical reactions that occur on the metal oxide surface.

In this work, we selected β-Ga_2_O_3_(100) as the model metal oxide surface and conducted systematic density functional theory (DFT) calculations. It was found that oxygen vacancy clusters can be readily formed on β-Ga_2_O_3_(100) upon reduction and that such vacancy clusters can stimulate the migration of hydrogen species from the surface into the near surface region. We then systematically studied H_2_ dissociation on three models of β-Ga_2_O_3_(100). These models represent a slightly reduced β-Ga_2_O_3_(100), a highly reduced β-Ga_2_O_3_(100), and a highly reduced β-Ga_2_O_3_(100) with near-surface hydrogen species. It was found that the concentration of oxygen vacancies has a negligible effect on the H_2_ dissociation. The preferred pathway in the two models without near-surface hydrogen species is homolytic dissociation of H_2_, which produces two hydrides. However, in the presence of near-surface hydrogen species, the other pathway featuring heterolytic dissociation and producing a hydride and a proton is selectively stabilized. This study also utilized CO_2_ hydrogenation as a probe reaction to gain further insight into the role of near-surface hydrogen species in catalysis. The investigation focused on the mechanism of key steps in the CO_2_ hydrogenation reaction, including CO_2_ adsorption and the initial hydrogenation step that produces HCOO or COOH species. Once again, it has been found that the presence of near-surface hydrogen species can tune the selectivity of surface chemical reactions, favoring the COOH pathway. This pathway is not favored on the other two models without near-surface hydrogen species.

## 2 Computational Details

All spin-polarized DFT calculations were performed using the *Vienna ab initio simulation package* (*VASP*).^26,27^ The Perdew–Burke–Ernzerhof (PBE)^28^ exchange–correlation functional under the generalized gradient approximation (GGA)^29^ was used in the calculations. The projector augmented wave (PAW)^30^ method was used to describe the valence–core interaction. The kinetic energy cutoff for the plane-waves was set at 400 eV, and the force threshold for structure optimizations was 0.05 eV/Å. The Brillouin-zone was sampled using *k*-point meshes of 5 × 3 × 1 and 4 × 2 × 1 for the bulk and surface calculations, respectively. Transition state (TS) structures were searched using the constrained minimization technique.^31^ Dispersion corrections were not considered on the basis that they have an insignificant effect on the adsorption energies of H and CO_2_ (see Tables S1 and S2).

The calculated lattice parameters for β-Ga_2_O_3_ (*a* = 12.45 Å, *b* = 3.08 Å, *c* = 5.88 Å, α = 90°, β = 103.68°, γ = 90°) agree well with the experimental values (*a* = 12.23 Å, *b* = 3.04 Å, *c* = 5.80 Å, α = 90°, β = 103.70°, γ = 90°).^32^ A 9-atomic-layer slab was used to model the β-Ga_2_O_3_(100) surface, extending at a (4 × 2) surface unit cell. The bottom three layers were fixed at their bulk positions in the calculations. A 15 Å vacuum layer was added along the *z*-axis to eliminate interactions between neighboring slabs.

The adsorption energies (*E*_ads_) were calculated by

1 $$\begin{array}{l} E_{\mathrm{ads}}=E_{x/\mathrm{sub}}-E_{\mathrm{sub}}-E_{x} \end{array}$$

where *E*_x/sub_, *E*_sub_, and *E*_x_ are the calculated total energies of the adsorption complex, the substrate, and the adsorbate. Note that for H adsorption, *E*_x_ represents half of the energy of a H_2_ molecule.

The oxygen vacancy formation energies (*E*_ov_) were calculated by

2 $$E_{\mathrm{ov}}=E_{\mathrm{vac}/\mathrm{sub}}+\frac{1}{2}E_{O_{2}}-E_{\mathrm{sub}}$$

where *E*_vac/sub_ and *E*_O_2__ are the calculated total energies of the substrate with one surface oxygen vacancy and a O_2_ molecule, respectively.

The relative population (*P*) of a given configuration *i* of H adsorption can be estimated using the free-energy-based Boltzmann distribution:

3 $$P_{i}=\frac{g_{i}\exp\left(-\frac{G_{i}}{k_{B}T}\right)}{\underset{j}{\sum }g_{j}\exp\left(-\frac{G_{j}}{k_{B}T}\right)}$$

where *g* represents the degeneracy factor, *G* is the Gibbs free energy, *i* and *j* are configurations, *k*_B_ is the Boltzmann constant, and *T* is temperature.

The Gibbs free energies of the H-adsorbed systems were calculated by

4 $$G=E_{\mathrm{DFT}}+E_{\mathrm{ZPE}}+\Delta U-TS_{\mathrm{vib}}$$

where *E*_DFT_, *E*_ZPE_, Δ*U*, and *S*_vib_ are the DFT-calculated total energy of the H-adsorbed system, the zero-point energy (ZPE) correction, the change in internal energy *U*, and the entropy due to vibrational motion, calculated using the harmonic oscillator approximation. The present treatment operates under the assumption that the pressure–volume work (*PV*) is negligible for solids.

## 3 Results and Discussion

### 3.1 Oxygen Vacancy Formation and Hydrogen Infiltration

In the presence of H_2_, surface O on β-Ga_2_O_3_(100) can react with H_2_, producing a H_2_O molecule and a surface oxygen vacancy. Our calculations show that a single surface oxygen vacancy (SV) on the β-Ga_2_O_3_(100) surface has a formation energy of 4.10 eV (see Figures 1a and S1). This SV provides an active site for subsequent H_2_ dissociation, which will be discussed in section 3.2. Additionally, SVs have the ability to migrate to deeper surface regions. This migration can be triggered by the fact that a subsurface oxygen vacancy has a formation energy that is 0.31 eV lower than that of a surface oxygen vacancy (see Figure S1). Further migration of a subsurface oxygen vacancy to the third oxygen layer from the β-Ga_2_O_3_(100) surface is also possible since the formation energy of a vacancy in the third oxygen layer is comparable to that of a surface oxygen vacancy (4.10 vs 4.11 eV, see Figure S1). The oxygen vacancy formation energy increases significantly by approximately 0.4 eV from the third oxygen layer to the fourth layer (see Figure S1). This makes it challenging for the oxygen vacancy to migrate toward the bulk region.

**Figure 1:**
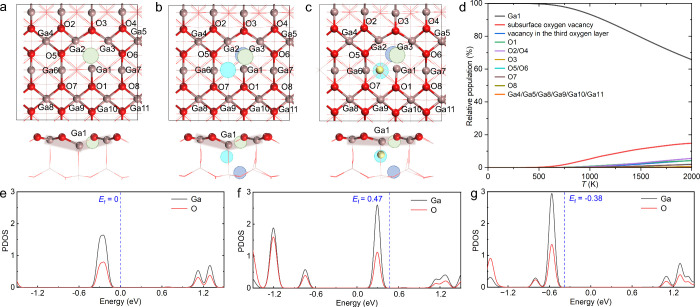
Calculated structures (top: top view; bottom: side view) of the (a) SV, (b) VC, and (c) VC+H models. The surface Ga, surface O, and near-surface H atoms are colored brown, red, and yellow, respectively. Atoms below the surface layer are shown in a line style. The positions of the surface oxygen vacancy, subsurface oxygen vacancy, and vacancy in the third oxygen layer are indicated by light green, cyan, and blue circles, respectively. This display scheme is used throughout the paper. The topographies of the surface Ga atoms of the SV, VC, and VC+H models are indicated by brown shadows. (d) Relative population of the configurations of H adsorption at various sites adjacent to or within the oxygen vacancy cluster in the VC model, including the surface sites (labeled in b), subsurface oxygen vacancy site, and the vacancy site in the third oxygen layer. Note that H is incapable of adsorbing at the Ga2 and Ga3 sites due to its strong binding with the neighboring Ga1 site. The calculated projected density of states (PDOS) of the Ga and O atoms adjacent to the surface oxygen vacancy (i.e., Ga1–Ga7 and O1–O8 as labeled in a, b, and c) in the (e) SV, (f) VC, and (g) VC+H models. The PDOS plots are aligned with respect to the lowest energy level of the 1s state of a fixed O atom at the bottom of the slabs.

Based on the findings presented above, we constructed a highly reduced model of the β-Ga_2_O_3_(100) surface that includes oxygen vacancy clusters (VCs). Each surface cell contains a VC composed of a surface oxygen vacancy, a subsurface oxygen vacancy, and a vacancy in the third oxygen layer (see Figure 1b). The calculated average vacancy formation energy of the VC is 3.99 eV, which is 0.11 eV lower than that of a SV. Therefore, the VC model (i.e., the β-Ga_2_O_3_(100) surface with VCs) is also representative, in addition to the SV model (i.e., the β-Ga_2_O_3_(100) surface with SVs). The results from these two models can be compared to study the impact of the vacancy concentration on surface chemical properties.

In the VC model, the oxygen vacancy clusters provide channels for the diffusion of surface hydrogen species toward deeper surface regions. Figure S2 shows the energy profile for the diffusion of the hydride species; the hydride species is initially adsorbed at the preferred Ga1 site and moves along the vacancy channel in the VC model. The calculated diffusion barriers (1.95 and 1.33 eV) are high, indicating that the near-surface hydrogen species formed are kinetically stable. Bader charge analyses confirmed that the near-surface hydrogen species are negatively charged (see Table S3), indicating that they are probably hydrides. Thermodynamically, the hydrogen species located in the subsurface oxygen vacancy and in the vacancy in the third oxygen layer are less stable than the surface hydride species by 0.32 and 0.54 eV, respectively. These energy increases are not considered significant. This finding is consistent with previous experimental and theoretical studies in that oxygen vacancies can facilitate the infiltration of hydrogen into metal oxides.^11,33,34^ The relative population of the configurations of H adsorption can be calculated from their Gibbs free energies and by assuming a Boltzmann distribution.^35–37^ It was found that the relative population of the configuration with H adsorbed at the subsurface oxygen vacancy (see the model in Figure 1c) increases significantly with increasing temperature, reaching a non-negligible value of approximately 5% at 800 K (see Figure 1d), a temperature typically employed for hydrogenation and dehydrogenation catalysis of Ga_2_O_3_.^1,19,38,39^ Therefore, it is possible for hydrogen species to exist in the near-surface region under catalytic conditions. Furthermore, it is important to note that a variety of experimental techniques and treatments have been demonstrated to be capable of producing H species in the near-surface region of metal oxides. These include Kelvin probe force spectroscopy (KPFS),^40,41^ collision-induced absorption,^42^ thermal annealing in H_2_,^43–45^ and plasma treatment.^46–48^

Accordingly, it is important to study the effect of near-surface hydrogen species on surface chemical reactions, and we thus developed a new model called VC+H, which is based on the VC model. The VC+H model differs from the VC model in that it includes an additional hydrogen species located in the subsurface oxygen vacancy (see Figure 1c). The VC+H model was developed based on two considerations. First, the hydrogen species is more likely to reside in the subsurface oxygen vacancy rather than the vacancy in the third oxygen layer. Second, subsurface hydrogen species can have a more significant impact on the surface chemical properties than hydrogen species in deeper layers.

### 3.2 H_2_ Dissociation

This section investigates alterations, if any, in the interaction between H_2_ and β-Ga_2_O_3_(100) upon deep reduction and the infiltration of surface hydrogen species. The dissociation of H_2_, which can produce reactive H species for both hydrogenation and dehydrogenation reactions, was studied. We first investigated this process on both reduced β-Ga_2_O_3_(100) surfaces. In principle, there are three pathways for H_2_ dissociation on those surfaces, including: (i) homolytic dissociation of H_2_ at the Ga···Ga site, generating two hydride species, (ii) heterolytic dissociation of H_2_ at the Ga–O site, generating a hydride and a proton, and (iii) homolytic dissociation of H_2_ at the O···O site, generating two protons.

To identify the optimal sites for H_2_ dissociation on the reduced β-Ga_2_O_3_(100) surfaces, we calculated the adsorption energies of H on different surface sites. Our calculations indicate that for both the SV and VC models H has a tendency to adsorb at the Ga site, forming a hydride species, rather than at the O sites, forming a proton (see Figure S3). The Ga1 site (labeled in Figures 1a and 1b) exhibits the highest affinity for H adsorption, with the SV and VC models producing H adsorption energies of −0.29 and −0.39 eV, respectively (see Figure S3). As expected, the VC model exhibits stronger binding with H due to the abundant excess electrons resulting from the formation of neutral oxygen vacancies (see Table S4). Furthermore, the highest occupied states of the VC model are distributed at higher energy levels than those of the SV model (see Figures 1e and 1f). Therefore, the VC model donates more electrons to the adsorbed H species, forming a more negatively charged hydride species (see Table S5), resulting in a more stable hydride species. This is consistent with a previous study^11^ that demonstrated an increase in the concentration of oxygen vacancies can stabilize surface hydride species.

As expected, pathway (iii) is unlikely because the final state with two protons has high energy, as indicated by its high reaction energy (see Figure S4). Given the equivalence of Ga2 and Ga3 in the SV model, the focus was exclusively on the homolytic dissociation of H_2_ on Ga1···Ga2. It is evident that, despite the nonequivalence of Ga2 and Ga3 in the VC model, the Ga1···Ga2 site exhibits a lower reaction energy than the Ga1···Ga3 site for the homolytic dissociation of H_2_ (see Table S6). Therefore, for the SV and VC models, we focus specifically on pathway (i) at the Ga1···Ga2 site and pathway (ii) at the Ga1–O1 site. For the SV model, pathway (i) has an activation barrier of 0.84 eV and an exergonic reaction energy of −0.26 eV; pathway (ii), on the other hand, has a higher activation barrier of 1.18 eV and an endergonic reaction energy of 0.30 eV (see Figure 2a). These findings are in line with the H adsorption results and suggest that H_2_ primarily undergoes homolytic dissociation in the SV model, leading to the formation of two hydride species at Ga sites surrounding the oxygen vacancy. The VC model shows a similar scenario (see Figure 2b). It was found that the concentration of the vacancies has a negligible effect on the activation barriers of H_2_ dissociation. However, it has a more pronounced effect on the stability of the products resulting from H_2_ dissociation. In particular, the product of the homolytic dissociation of H_2_ dissociation (i.e., two hydride species) is less stable in the VC model than in the SV model. This is likely due to the stronger electrostatic repulsion between the two adjacent hydride species in the VC model compared to those in the SV model (see Figures 2d and 2e).

**Figure 2:**
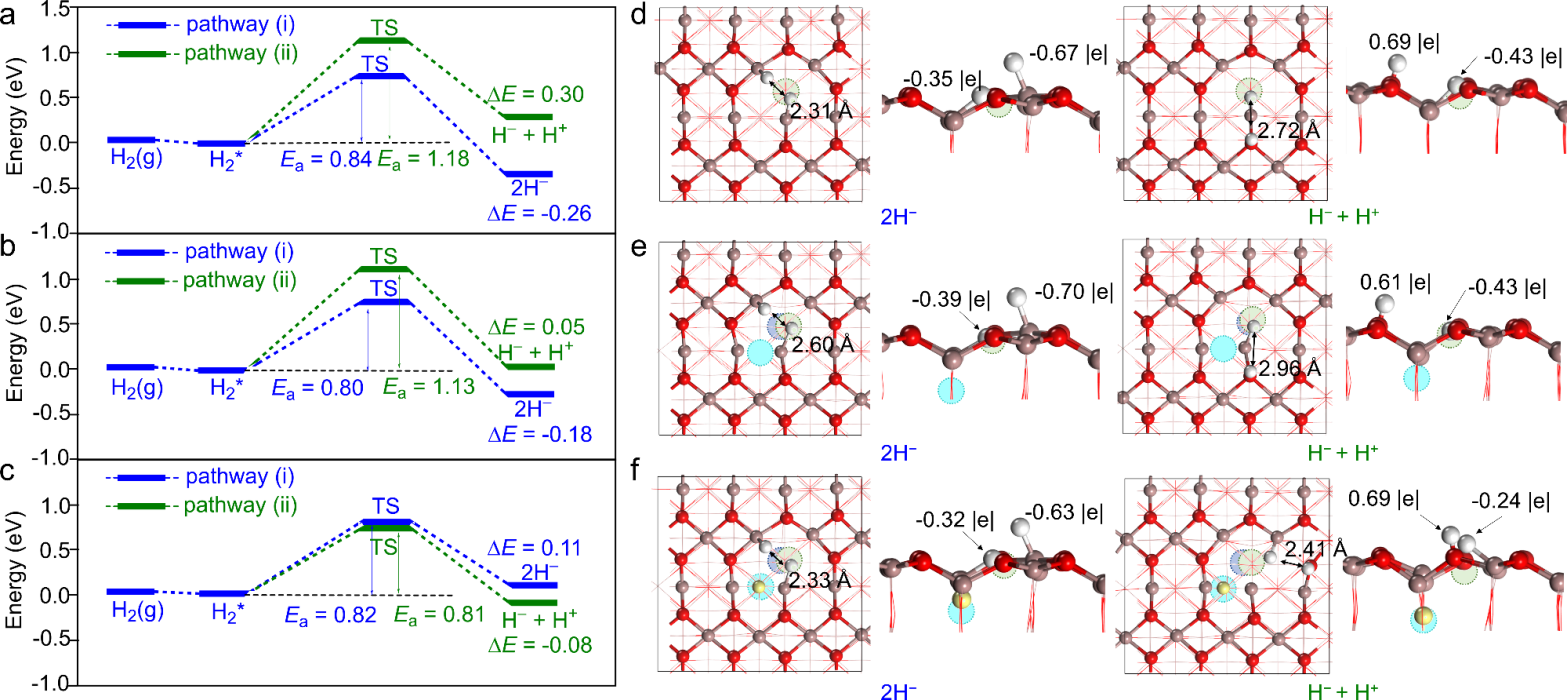
Calculated energy profiles for H_2_ dissociation through pathways (i) and (ii) in the (a) SV, (b) VC, and (c) VC+H models. The states H_2_(*g*), H_2_*, 2H^–^, and H^–^ + H^+^ represent the states with a gas-phase H_2_, an adsorbed H_2_, two hydride species, and one hydride plus one proton species, respectively. TS represents the transition state of H_2_ dissociation. *E*_a_ and Δ*E* represent activation barrier and reaction energy, respectively. Calculated structures (left: top view; right: side view) of the products of H_2_ dissociation (i.e., the states 2H^–^ and H^–^ + H^+^) in the (d) SV, (e) VC, and (f) VC+H models. The calculated Bader charges of the surface H species (depicted in white) and the distances between them are also given.

In the VC+H model, the Ga1 site is once again identified as the optimal location for H adsorption. However, the presence of near-surface H species results in repulsive interactions, thereby causing the H species to point toward Ga3. Consequently, the Ga1···Ga2, Ga1···Ga3, and Ga2···Ga3 sites were considered for homolytic dissociation of H_2_, and the Ga1–O1, Ga3–O3, Ga3–O4, and Ga3–O6 sites for heterolytic dissociation of H_2_. Table S6 shows that the Ga1···Ga2 site exhibits the lowest reaction energy for homolytic dissociation of H_2_, and heterolytic dissociation is observed to preferentially occur at the Ga3–O6 site. Intriguingly, the VC+H model presents a different scenario for H_2_ dissociation compared to the SV and VC models (see Figure 2c). According to the VC+H model, the heterolytic dissociation of H_2_ through pathway (ii) is more favorable than the homolytic dissociation through pathway (i); pathway (ii) has an activation barrier of 0.81 eV and a reaction energy of −0.08 eV, while pathway (i) has an activation barrier of 0.82 eV and a reaction energy of 0.11 eV. It is important to note that the heterolytic dissociation, which is endergonic in the SV and VC models, becomes exergonic in the VC+H model. In the presence of near-surface hydrogen species, the heterolytic dissociation product (one hydride and one proton; see Figure 2f) is 0.19 eV more stable than the homolytic dissociation product (two hydrides; see Figure 2f). This finding contradicts the predictions of the SV and VC models, which suggest that the homolytic dissociation product is more stable than the heterolytic dissociation product. Bader charge analyses indicate that introducing near-surface hydrogen species into the VC model results in the modulation of the surface electronic properties. This leads to a more positive charge on the adjacent Ga atoms to the surface oxygen vacancy, while the adjacent O atoms become more negatively charged (see Figure 3 and Table S4). As a result, the polarization of surface Ga–O bonds increases in the VC+H model compared with the SV and VC models, which promotes the heterolytic dissociation of H_2_. In addition, near-surface hydrogen species shift the highest occupied states of the Ga and O atoms adjacent to the surface oxygen vacancy to lower energy levels (see Figures 1e-1g). As a result, the VC+H model has a weak electron-donating capability, making it difficult to dissociate H_2_ homolytically to generate two hydrides. The results suggest that near-surface hydrogen species can modify the surface electronic and chemical properties, which in turn can tune the selectivity of surface chemical reactions. It is important to note that it is difficult for surface hydrogen species to produce effects similar to those produced by near-surface hydrogen species. This is because surface hydrogen species either block the active sites for the heterolytic dissociation of H_2_ (see Figure S5a) or have a negligible effect on the electronic properties of the active sites (see Figures S5b-S5j).

**Figure 3:**
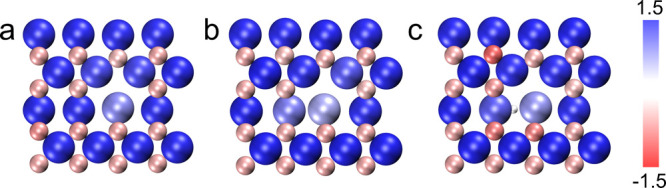
Surface charge distributions of the (a) SV, (b) VC, and (c) VC+H models. Big and small balls represent Ga and O atoms, respectively. Blue and red atoms are positively and negatively charged, respectively. The values of the charges are represented by different colors.

### 3.3 CO_2_ Hydrogenation

The CO_2_ hydrogenation reaction was selected as the second test for this study. We then studied this reaction in the hydrogenated SV, hydrogenated VC, and hydrogenated VC+H models; the preadsorbed H species provide sources of H for the hydrogenation of CO_2_. To account for the preference of homolytic dissociation of H_2_ to produce hydrides in the SV and VC models, we considered a preadsorbed hydride species on these two models to construct the corresponding hydrogenated models (denoted as H^–^/SV and H^–^/VC, respectively, see Figures 4a and 4b). We considered two cases for the hydrogenated VC+H model: one with a preadsorbed hydride species on the surface (denoted as H^–^/VC+H, see Figure 4c) and the other with a preadsorbed proton species on the surface (denoted as H^+^/VC+H, see Figure 4d). This consideration is based on the finding that heterolytic dissociation of H_2_ to produce one hydride and one proton is preferred in the VC+H model (see Figure 2c).

**Figure 4:**
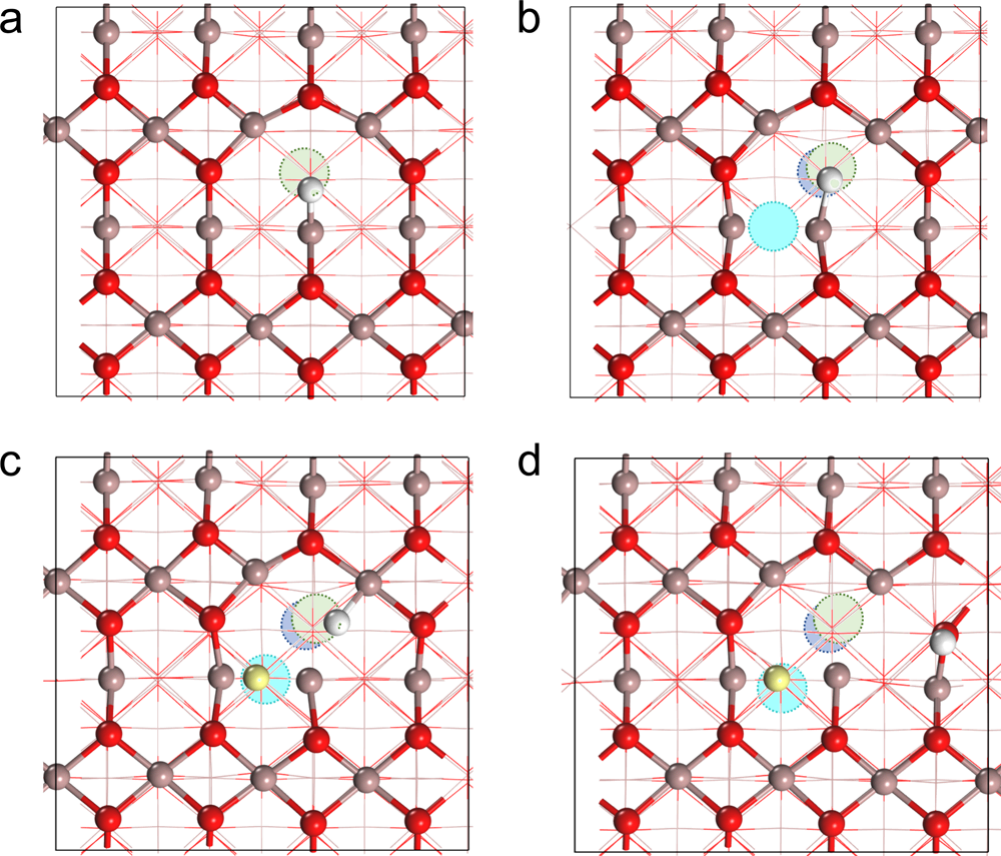
Calculated structures of (a) H^–^/SV, (b) H^–^/VC, (c) H^–^/VC+H, and (d) H^+^/VC+H models.

The adsorption of CO_2_ and the subsequent first hydrogenation reaction are widely recognized as crucial steps in CO_2_ hydrogenation.^49,50^ The selectivity of CO_2_ hydrogenation is largely determined by the two products of the first hydrogenation step, namely HCOO and COOH.^51–54^ Therefore, we focus primarily on these two steps. We found that CO_2_ tends to be adsorbed linearly in the H^–^/SV, H^–^/VC, and H^–^/VC+H models (see Figures 5a-5c), while bent CO_2_ is preferred in the H^+^/VC+H model (see Figure 5d); the calculated most favorable adsorption energies of CO_2_ in the H^–^/SV, H^–^/VC, H^–^/VC+H, and H^+^/VC+H models are −0.05, −0.03, −0.04, and −0.93 eV, respectively (see Table S7). In the case of the H^+^/VC+H model, electrons are injected from the adsorbed H on O to the nearby Ga atoms surrounding the oxygen vacancy, making those Ga atoms electron-rich (see Table S5). As a result, CO_2_ can be effectively activated in the H^+^/VC+H model by withdrawing electrons from the Ga atoms, resulting in significantly stronger binding with the surface (see Table S7).

**Figure 5:**
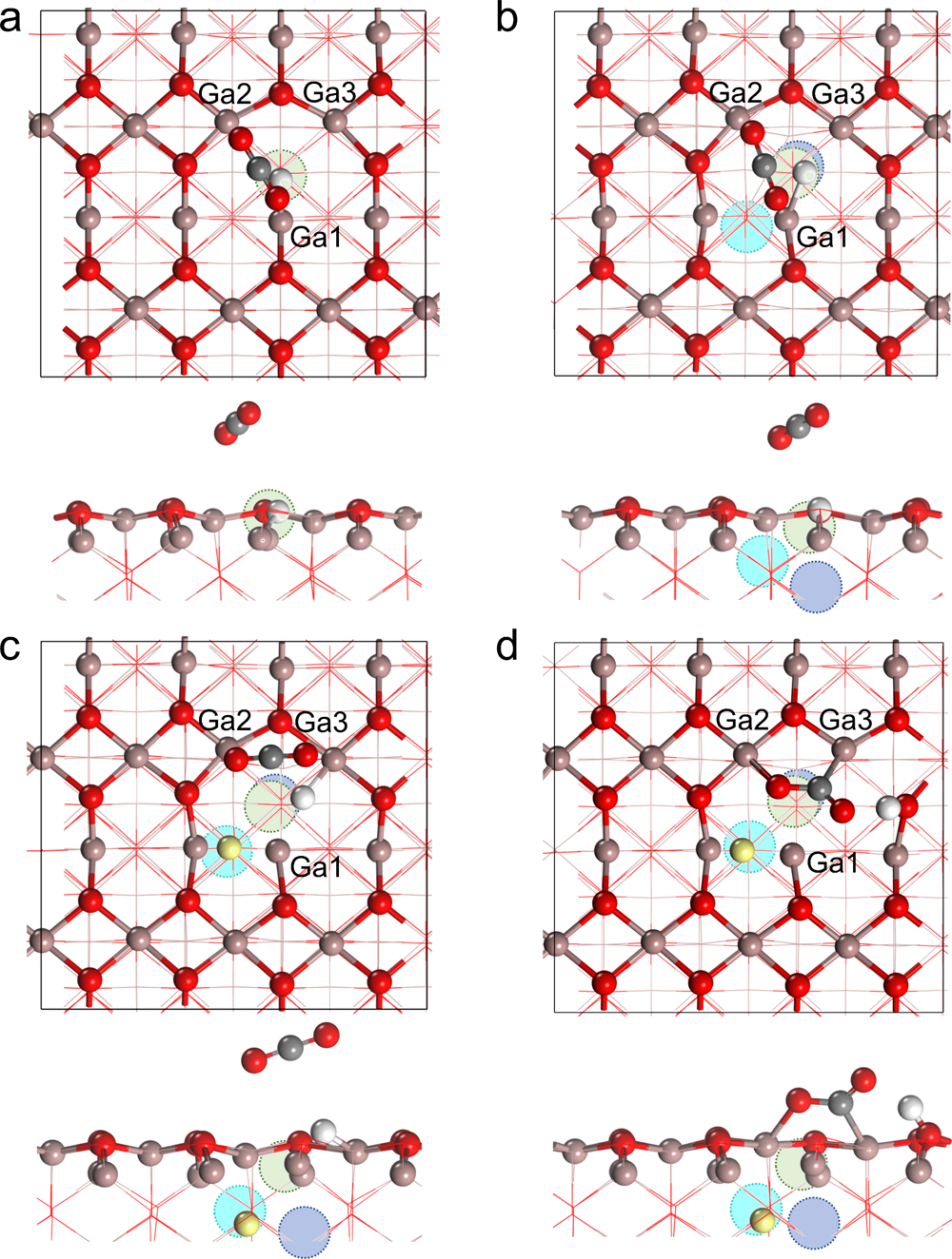
Calculated most favorable configuration (top: top view; bottom: side view) of CO_2_ adsorption in the (a) H^–^/SV, (b) H^–^/VC, (c) H^–^/VC+H, and (d) H^+^/VC+H models. C atoms are depicted in gray.

We then studied the hydrogenation of CO_2_ to COOH and HCOO species in the H^–^/SV, H^–^/VC, H^–^/VC+H, and H^+^/VC+H models. The HCOO pathway is expected to dominate in the H^–^/SV model due to its much lower activation barrier and reaction energy compared to the COOH pathway (see Figure 6a); this finding is not surprising since hydride species tend to attack the positively charged carbon atom of CO_2_ rather than the negatively charged oxygen. The calculated activation barrier and reaction energy for hydrogenation of CO_2_ to HCOO in the H^–^/SV model are 0.55 and 0.41 eV, respectively. The H^–^/VC model produced similar results to that of the H^–^/SV model (see Figure 6b). This is likely due to the sufficient electron-donating capability of the H^–^/SV model in the formation of the negatively charged HCOO and COOH species, resulting in comparable charge transfer results for the H^–^/SV and H^–^/VC models (see Table S8). The H^–^/VC+H model yielded a calculated activation barrier and reaction energy of 0.58 and −0.18 eV, respectively, for CO_2_ hydrogenation to HCOO (see Figure 6c). Interestingly, the COOH pathway is notably more facile in the H^–^/VC+H model (activation barrier: 0.89 eV; reaction energy: 0.48 eV) compared to H^–^/SV (activation barrier: 1.75 eV; reaction energy: 1.12 eV) and H^–^/VC (activation barrier: 1.70 eV; reaction energy: 0.93 eV). Thus, the selectivity for COOH is expected to increase significantly on the basis of the H^–^/VC+H model. The change in the energy profiles for the H^–^/VC+H model can also be attributed to the increased polarization of surface Ga–O bonds caused by the presence of near-surface hydrogen species. Specifically, the charges of the formed HCOO and COOH species in the VC+H model are more negative compared to those of the corresponding species in the SV and VC models. Moreover, the bonds in the HCOO and COOH species in the VC+H model are more polarized than those in the SV and VC models, indicating a strong interaction between HCOO/COOH and the VC+H model (see Table S8). Nevertheless, the COOH pathway in the H^–^/VC+H model is more significantly stabilized than the HCOO pathway. Not surprisingly, the reaction of CO_2_ and H^+^ in the H^+^/VC+H model to produce COOH is facile, with a calculated activation barrier and reaction energy of 0.13 and 0.06 eV, respectively (see Figure 6d). In contrast, the reaction of CO_2_ and H^+^ to produce HCOO is hindered due to the high activation barrier of 1.11 eV (see Figure 6d). These results suggest that the SV and VC models selectively produce HCOO, while the VC+H model can significantly enhance the selectivity for COOH. These findings further emphasize the role of near-surface hydrogen species in tuning the selectivity of surface chemical reactions.

**Figure 6:**
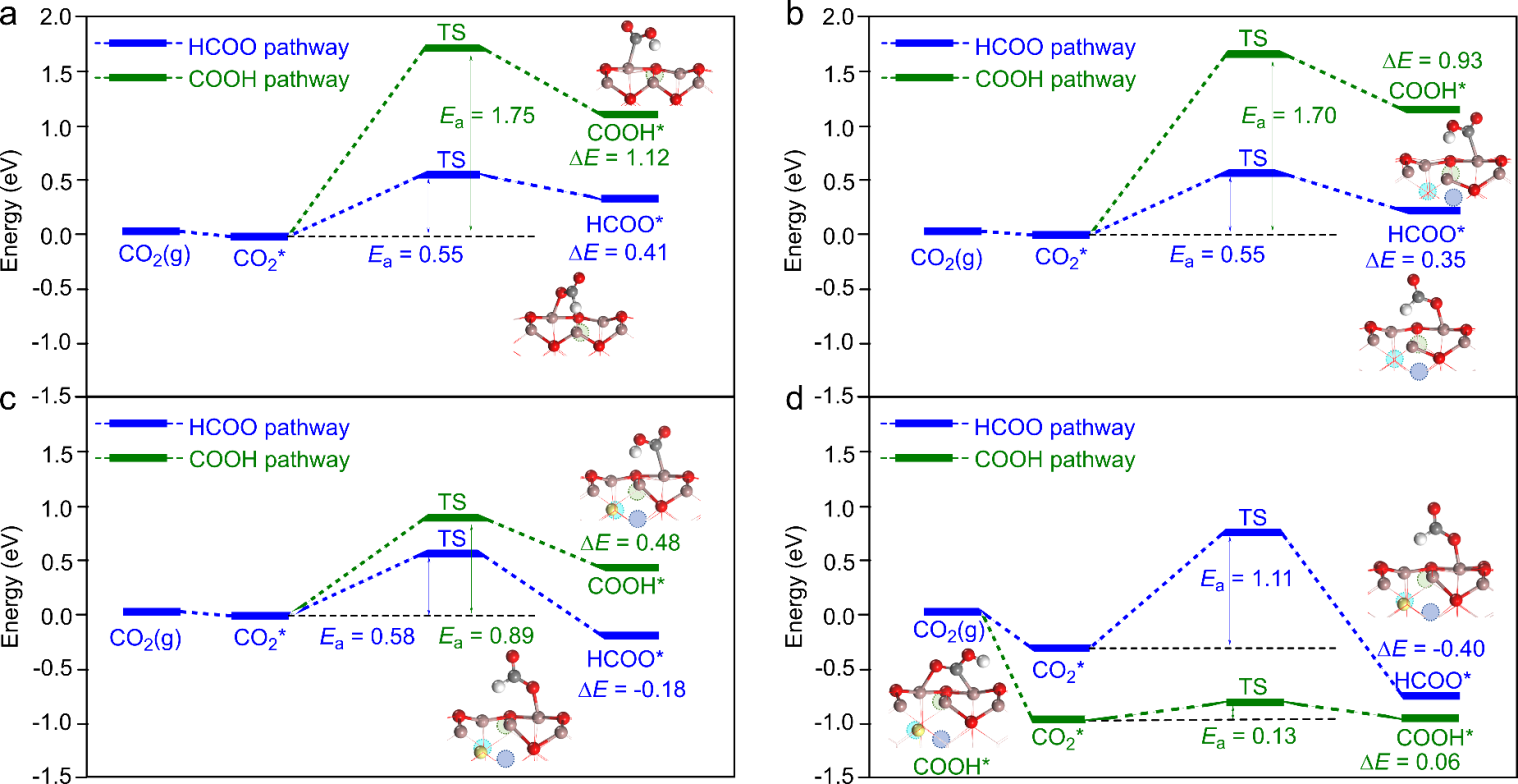
Calculated energy profiles for the adsorption of CO_2_ and the subsequent first hydrogenation reaction through the HCOO and COOH pathways in the (a) H^–^/SV, (b) H^–^/VC, (c) H^–^/VC+H, and (d) H^+^/VC+H models. The states CO_2_(*g*), CO_2_*, HCOO*, and COOH* represent the states with a gas-phase CO_2_, an adsorbed CO_2_, a HCOO species, and a COOH species, respectively. TS represents the transition state of the first hydrogenation reaction. *E*_a_ and Δ*E* represent activation barrier and reaction energy, respectively. The corresponding structures are listed in Figure S6.

It is important to mention that the results of the present study are in substantial agreement with those of a previous experimental investigation by Castro-Fernández and co-workers on propane dehydrogenation over a Ga_2_O_3_ catalyst.^55^ They found that H_2_ treatment resulted in the increase of the oxygen vacancy concentration, both at the surface and within the bulk of Ga_2_O_3_. Furthermore, H_2_-treated Ga_2_O_3_ exhibited a surface structure that was found to be “more ordered” compared to the untreated sample. This is consistent with our finding that the presence of near-surface hydrogen species induces an outward displacement of the Ga1 atom, leading to a more ordered surface structure of VC+H compared to that of SV and VC having no near-surface hydrogen species (see Figures 1a-1c). They also found that H_2_-treated Ga_2_O_3_ demonstrated a substantial alteration in product selectivity. It is likely that such selectivity alteration is partially contributed by near-surface hydrogen species, as indicated by theoretical calculations. It is important to note that, to the best of our knowledge, this novel mechanism – namely, the near-surface hydrogen species-induced selectivity change of chemical reactions on metal oxide surfaces – has not been identified in previous studies. Consequently, the findings of this study offer a new perspective on H_2_-involved metal oxide catalysis, and we believe that this development is of considerable significance in the research field of metal oxide catalysis.

## 4 Conclusion

In this study, we first investigated the formation of oxygen vacancies on the surface and in the near-surface regions of β-Ga_2_O_3_(100). Our results show that both single vacancies and vacancy clusters can form on β-Ga_2_O_3_(100) upon reduction. Additionally, our calculations suggest that the vacancy cluster allows hydrogen species to reside in the near-surface region of β-Ga_2_O_3_(100), particularly under the typical experimental conditions for catalyzing hydrogenation and dehydrogenation reactions on the Ga_2_O_3_ catalyst surfaces. We then studied the effect of deep reduction and the infiltration of surface hydrogen species on the dissociation of H_2_ on β-Ga_2_O_3_(100). The results show that H_2_ dissociates homolytically to produce two hydrides on both slightly and highly reduced β-Ga_2_O_3_(100) (represented by the single vacancy model and the vacancy cluster model, respectively). However, the presence of near-surface hydrogen species results in modulation of the surface electronic properties and alteration of the preferred pathway to heterolytic dissociation, leading to the formation of a hydride and a proton species. CO_2_ hydrogenation was selected as another example to clarify the impact of near-surface hydrogen species on surface reactivity. We found that the presence of near-surface hydrogen species considerably improves the selectivity of the COOH pathway over the HCOO pathway in CO_2_ hydrogenation. The study suggests that near-surface hydrogen species can have a significant impact on the surface chemical properties of metal oxides, which, in turn, can tune the selectivity of surface chemical reactions. This finding is largely consistent with previously reported experimental results,^55^ and notably, it provides an alternative yet highly probable mechanism to understand the experimental findings. Future research should seriously consider the effect of near-surface hydrogen species on the surface chemistry of metal oxides.

## Supplementary Material



## Notes

Vacancy formation energies, H adsorption energies, energy profile for H diffusion, H_2_ dissociation energies, and corresponding structures and Bader charges (PDF)
