# Supplementary material for: Near-Surface Hydrogen Species Tune the Selectivity of Chemical Reactions on Metal Oxide Surfaces
Source: Precis Chem. 2025 Aug 1;3(12):777–85. doi: 10.1021/prechem.5c00060 (PMC12728764; doi:10.1021/prechem.5c00060)
Supplement: Supplementary file 1 [file pc5c00060_si_001.pdf]

Supporting Information

Near-Surface Hydrogen Species Tunes the Selectivity of Chemical Reactions on Metal Oxide Surfaces

Yi-Chun Chu,[1] Weixin Huang,[2]* Xin-Ping Wu,[1]* and Xue-Qing Gong[3]

[1]State Key Laboratory of Green Chemical Engineering and Industrial Catalysis, Centre for Computational Chemistry and Research Institute of Industrial Catalysis, School of Chemistry and Molecular Engineering, East China University of Science and Technology, Shanghai 200237, P.R. China.

[2]State Key Laboratory of Precision and Intelligent Chemistry, *i*ChEM, Key Laboratory of Surface and Interface Chemistry and Energy Catalysis of Anhui Higher Education Institutes, Department of Chemical Physics, University of Science and Technology of China, Hefei 230026, P.R. China.

[3]State Key Laboratory of Synergistic Chem-Bio Synthesis, School of Chemistry and Chemical Engineering, Shanghai Jiao Tong University, Shanghai 200240, P.R. China.

*Corresponding authors:

E-mail: huangwx@ustc.edu.cn (W.H.)

E-mail: xpwu@ecust.edu.cn (X.-P.W.)

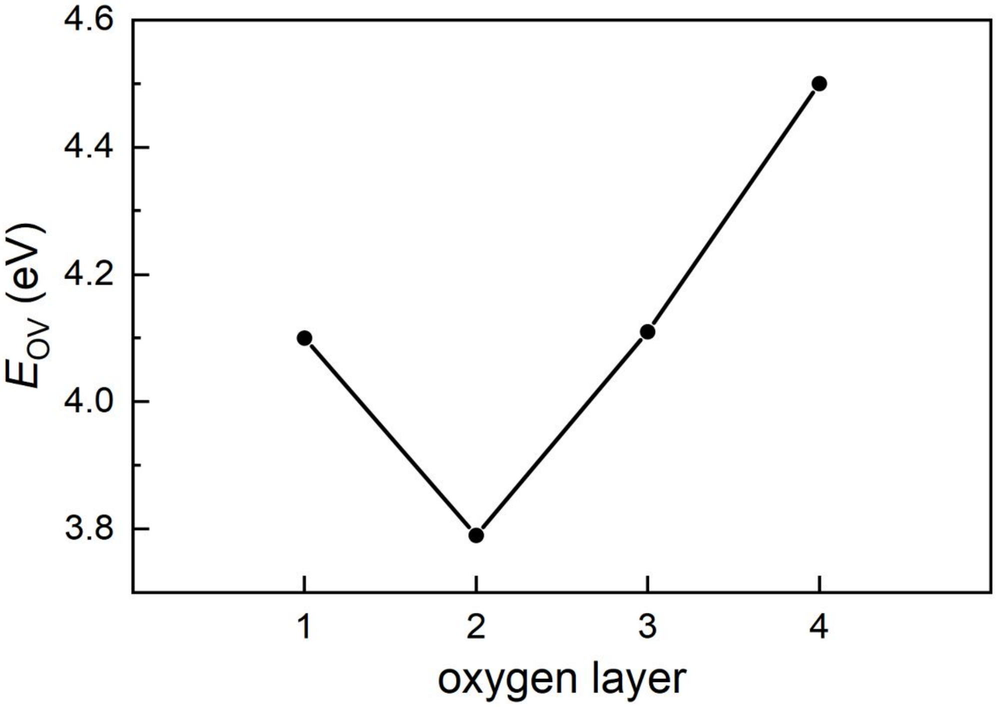

Figure S1. Calculated formation energies of vacancies in different oxygen layers from the β-Ga_2O_3(100) surface.

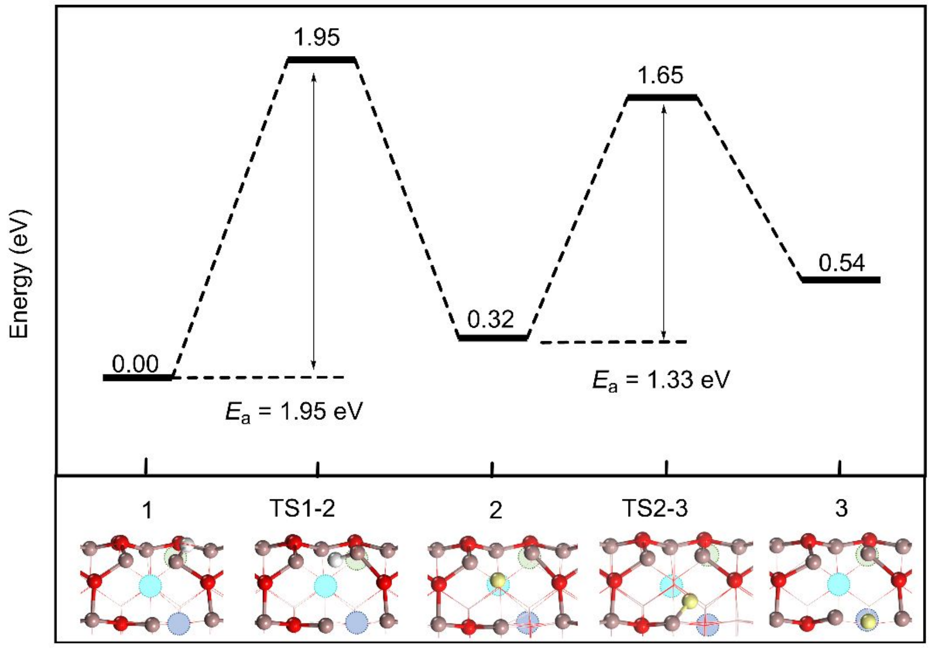

Figure S2. Calculated energy profile for the diffusion of hydride species from the surface Ga1 site to the deeper surface regions of the VC model.

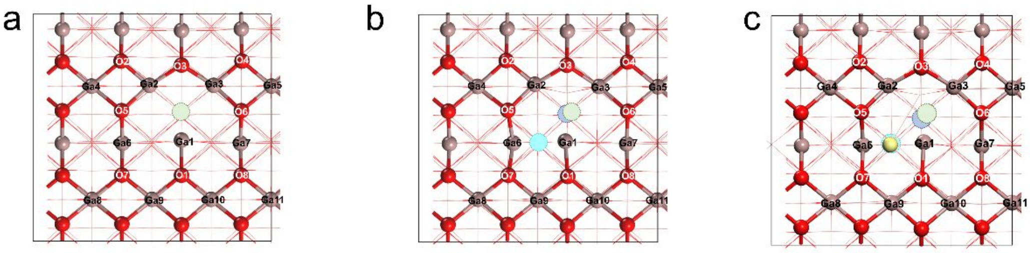

(a)	(b)	(c)
E_{ads}(Ga1) = -0.29 eV	E_{ads}(Ga1) = -0.39 eV	E_{ads}(Ga1) = -0.07 eV
E_{ads}(Ga4) = 1.20 eV	E_{ads}(Ga4) = 1.17 eV	E_{ads}(Ga4) = 1.28 eV
E_{ads}(O1) = 0.20 eV	E_{ads}(O1) = -0.14 eV	E_{ads}(O1) = 0.31 eV
E_{ads}(O2) = 0.06 eV	E_{ads}(O2) = 0.03 eV	E_{ads}(O2) = 0.34 eV
E_{ads}(O3) = 0.61 eV	E_{ads}(O3) = 0.22 eV	E_{ads}(O3) = 0.62 eV
E_{ads}(O4) = 0.06 eV	E_{ads}(O4) = 0.03 eV	E_{ads}(O4) = 0.33 eV
E_{ads}(O5) = 0.21eV	E_{ads}(O5) = 0.11eV	E_{ads}(O5) = 0.42eV
E_{ads}(O6) = 0.21 eV	E_{ads}(O6) = 0.10 eV	E_{ads}(O6) = 0.46 eV
E_{ads}(O7) = -0.01 eV	E_{ads}(O7) = -0.01 eV	E_{ads}(O7) = 0.30 eV
E_{ads}(O8) = 0.20 eV	E_{ads}(O8) = 0.10 eV	E_{ads}(O8) = 0.32 eV

Figure S3. Calculated adsorption energies (E_{ads}) of H at different surface sites of the (a) SV, (b) VC, and (c) VC+H models.

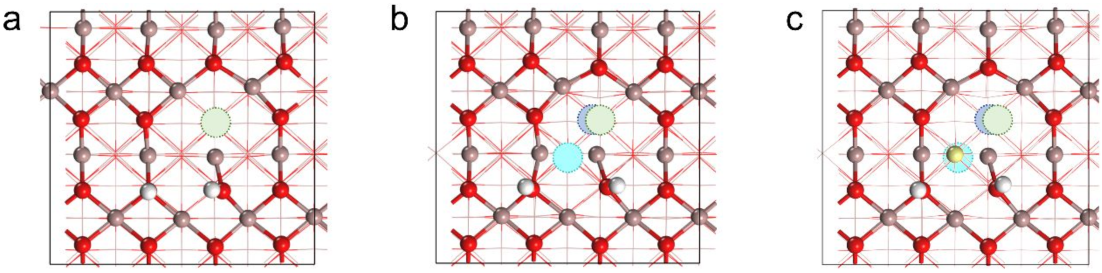

ΔE = 0.60 eV | ΔE = 0.38 eV | ΔE = 0.47 eV

Figure S4. Calculated reaction energies (ΔE) and structures of the products for homolytic dissociation of H_2 at the O…O site of the (a) SV, (b) VC, and (c) VC+H models.

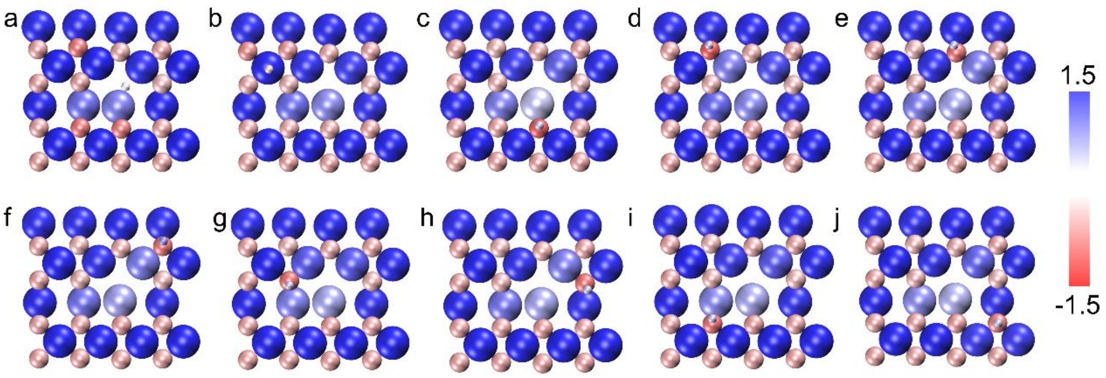

Figure S5. Surface charge distributions of the hydrogen-adsorbed VC models with hydrogen species adsorbed on (a) Ga1, (b) Ga4, (c) O1, (d) O2, (e) O3, (f) O4, (g) O5, (h) O6, (i) O7, or (j) O8. Big and small balls represent Ga and O atoms, respectively. Blue and red atoms are positively charged and negatively charged, respectively. The values of the charges are represented by different colors.

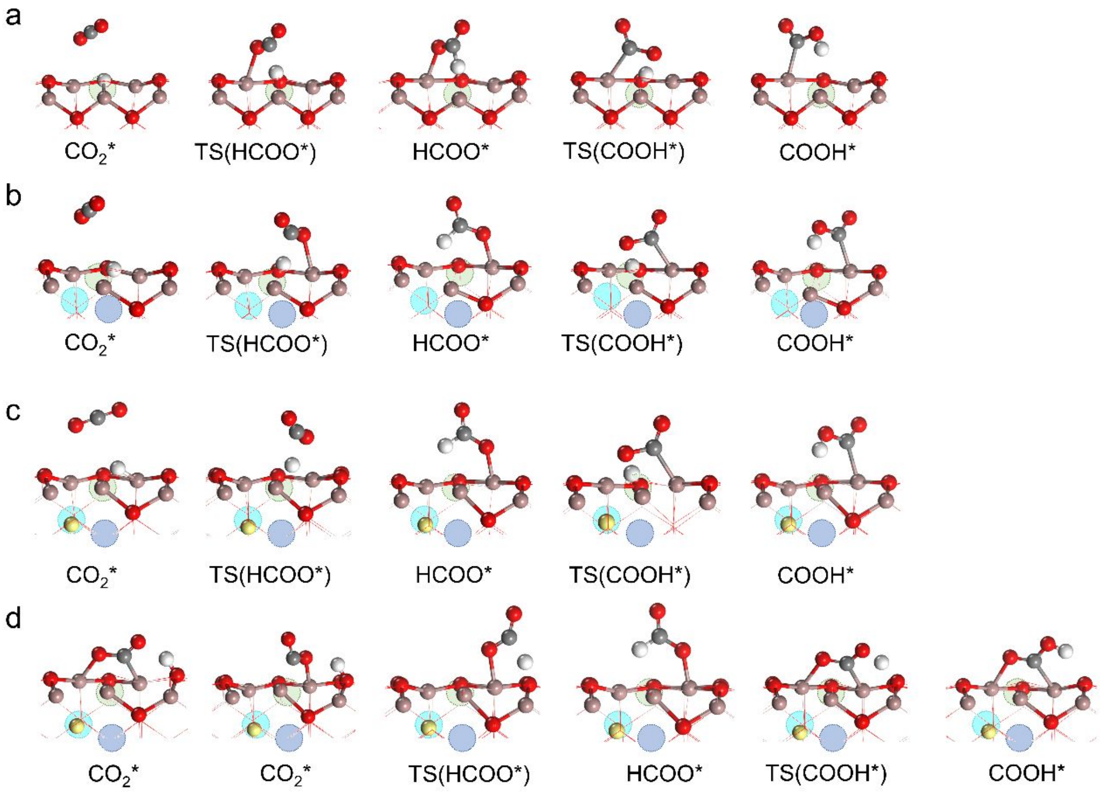

Figure S6. Calculated structures for the adsorption of CO_2 and the subsequent first hydrogenation reaction through the HCOO and COOH pathways on the (a) H^-/SV, (b) H^-/VC, (c) H^-/VC+H, and (d) H^+/VC+H models.

Table S1. Calculated most favorable adsorption energies (in eV) of H on SV, VC, and VC+H by DFT and DFT-D3 methods, and their differences (the DFT value minus the DFT-D3 value)

	SV	VC	VC+H
DFT	−0.29	−0.39	−0.07
DFT-D3	−0.30	−0.40	−0.08
difference	0.01	0.01	0.01

Table S2. Calculated most favorable adsorption energies (in eV) of CO_2 on H^-/SV, H^-/VC, H^-/VC+H, and H^+/VC+H by DFT and DFT-D3 methods, and their differences (the DFT value minus the DFT-D3 value)

	H^-/SV	H^-/VC	H^-/VC+H	H^+/VC+H
DFT	−0.05	−0.03	−0.04	−0.93
DFT-D3	−0.09	−0.07	−0.08	−0.99
difference	0.04	0.04	0.04	0.06

Table S3**.** Calculated Bader charges (|e|) of the surface hydride species and the hydrogen species located in the subsurface oxygen vacancy and in the vacancy in the third oxygen layer

oxygen layer	first (surface)	second (subsurface)	third
Bader charge	−0.29	−0.14	−0.09

Table S4. Calculated Bader charges (|e|) of the Ga and O atoms adjacent to the surface oxygen vacancy (i.e., Ga1−Ga7 and O1−O8 as labelled in Figure S3) on the SV, VC, and VC+H models

	SV	VC	VC+H
**Ga site**			
Ga1	0.86	0.68	0.71
Ga2	1.54	1.58	1.59
Ga3	1.54	1.48	1.59
Ga4	1.67	1.68	1.71
Ga5	1.67	1.66	1.67
Ga6	1.64	1.10	1.47
Ga7	1.64	1.64	1.62
sum	10.56	9.82	10.36
**O site**			
O1	−1.12	−1.10	−1.14
O2	−1.15	−1.13	−1.23
O3	−1.05	−1.11	−1.09
O4	−1.15	−1.13	−1.14
O5	−1.13	−1.10	−1.14
O6	−1.13	−1.10	−1.10
O7	−1.10	−1.12	−1.16
O8	−1.12	−1.11	−1.11
sum	−8.95	−8.90	−9.11

Table S5. Calculated Bader charges (|e|) of the Ga sites surrounding the surface oxygen vacancy (i.e., Ga1−Ga3 as labelled in Figure S3) and the surface adsorbed hydrogen species on the H^-/SV, H^-/VC, H^-/VC+H, and H^+/VC+H models

	Ga1	Ga2	Ga3	H^-	H^+
H^-/SV	1.46	1.64	1.64	−0.18	-
H^-/VC	0.98	1.65	1.59	−0.29	-
H^-/VC+H	0.84	1.67	1.55	−0.43	-
H^+/VC+H	0.73	1.64	1.07	-	0.62

Table S6. Calculated reaction energies (in eV) for homolytic dissociation of H_2 at different Ga…Ga sites and heterolytic dissociation of H_2 at different Ga−O sites on the SV, VC and VC+H models

	SV	VC	VC+H
**homolytic**			
Ga1…Ga2	−0.26	−0.18	0.11
Ga1…Ga3	-	0.01	0.26
Ga2…Ga3	-	-	0.24
**heterolytic**			
Ga1−O1	0.30	0.05	0.14
Ga3−O3	-	-	0.27
Ga3−O4	-	-	0.20
Ga3−O6	-	-	−0.08

Table S7. Calculated most favorable adsorption energies (E_{ads}, in eV) of CO_2 on the H^-/SV, H^-/VC, H^-/VC+H, and H^+/VC+H models and the calculated Bader charges (|e|) of the adsorbed CO_2 molecules

	H^-/SV	H^-/VC	H^-/VC+H	H^+/VC+H
E_{ads}	−0.05	−0.03	−0.04	−0.93
Bader charge	−0.03	−0.01	−0.01	−1.06

Table S8. Calculated Bader charges (|e|) of the products (HCOO and COOH) of the first hydrogenation step of CO_2 hydrogenation on the H^-/SV, H^-/VC, and H^-/VC+H models and the individual atoms in the HCOO and COOH species

	H^-/SV		H^-/VC		H^-/VC+H	
	HCOO	COOH	HCOO	COOH	HCOO	COOH
total	−0.60	−0.35	−0.61	−0.39	−0.72	−0.41
C	1.62	1.21	1.53	1.13	1.75	1.24
O	−1.10	−1.22	−0.95	−1.43	−1.13	−1.45
O	−1.05	−0.91	−1.10	−0.73	−1.31	−0.78
H	−0.07	0.57	−0.09	0.64	−0.03	0.58
